# Monitoring Physical Activity with a Wearable Sensor in Patients with COPD during In-Hospital Pulmonary Rehabilitation Program: A Pilot Study

**DOI:** 10.3390/s21082742

**Published:** 2021-04-13

**Authors:** Sebastian Rutkowski, Joren Buekers, Anna Rutkowska, Błażej Cieślik, Jan Szczegielniak

**Affiliations:** 1 Faculty of Physical Education and Physiotherapy, Opole University of Technology, 45-758 Opole, Poland; a.rutkowska@po.edu.pl (A.R.); j.szczegielniak@po.edu.pl (J.S.); 2 Department of Biosystems, KU Leuven, 3001 Leuven, Belgium; joren.buekers@kuleuven.be; 3 ISGlobal, 08002 Barcelona, Spain; 4 Universitat Pompeu Fabra (UPF), 08002 Barcelona, Spain; 5 CIBER Epidemiología y Salud Pública (CIBERESP), 08002 Barcelona, Spain; 6 Faculty of Health Sciences, Jan Dlugosz University in Czestochowa, 42-200 Czestochowa, Poland; b.cieslik@ajd.czest.pl

**Keywords:** COPD, wearable sensors, SenseWear Armband, physical activity, weekday-to-weekend, energy expenditure

## Abstract

Accelerometers have become a standard method of monitoring physical activity in everyday life by measuring acceleration in one, two, or three axes. These devices provide reliable and objective measurements of the duration and intensity of physical activity. We aimed to investigate whether patients undertake physical activity during non-supervised days during stationary rehabilitation and whether patients adhere to the rigor of 24 h monitoring. The second objective was to analyze the strengths and weaknesses of such kinds of sensors. The research enrolled 13 randomly selected patients, qualified for in-patient, 3 week, high-intensity, 5 times a week pulmonary rehabilitation. The SenseWear armband was used for the assessment of physical activity. Participants wore the device 24 h a day for the next 4 days (Friday–Monday). The analysis of the number of steps per day, the time spent lying as well as undertaking moderate or vigorous physical activity (>3 metabolic equivalents of task (METs)), and the energy expenditure expressed in kcal showed no statistically significant difference between the training days and the days off. It seems beneficial to use available physical activity sensors in patients with chronic obstructive pulmonary disease (COPD); measurable parameters provide feedback that may increase the patient’s motivation to be active to achieve health benefits.

## 1. Introduction

Chronic obstructive pulmonary disease (COPD) is a progressive disease that limits airflow through the respiratory tract. It is estimated that the disease affects 210 million people worldwide [1]. COPD is a leading cause of morbidity and mortality worldwide and will become the fourth leading cause of death by 2030. The Global Initiative for Chronic Obstructive Lung Disease (GOLD) defines COPD as a disease state characterized by airflow limitation, causing shortness of breath and significant systemic effects involving the lung and likewise causing extrapulmonary adverse reactions, with a high disease rate, high disability rate, high mortality rate, and a long course of disease [2]. The occurrence of pain in the cervical and thoracic spine region is very common, this probably leads to changes in the muscle tone [3]. COPD has also been shown to impair coordination and reduce balance and agility. In comparison to healthy people, patients with COPD demonstrate significant deficiencies in performing motor tasks, as well as in postural balance [4]. COPD is characterized not only by shortness of breath, dyspnea, chronic cough, and sputum production but also by fatigue and reductions in both physical capacity and physical activity [5,6]. A study by Theander and Unosson reported that patients with COPD perceived significantly greater functional limitations in cognitive, physical, and psychosocial functioning due to fatigue compared to those in a control group [7]. The prevalence of the symptom is high; in a study concerning the severity of fatigue in patients with stable, moderate-to-severe COPD, it was shown that almost half of all patients experienced abnormal fatigue: 23% mild fatigue and 24% severe fatigue [8]. Fatigue affected even greater proportions of patients than either depression or anxiety [9]. The negative effect of fatigue on the patient’s daily life is manifested in many aspects. Individuals indicated that physical limitations were mainly focused on walking and moving and performing homework, and personal hygiene was sometimes too physically demanding. All these symptoms cause a limitation of the level of physical activity, which in turn causes deterioration of physical health.

The level of physical activity in patients with COPD is, therefore, lower than that in healthy individuals with respect to age [10,11] and lower than that in individuals with other chronic conditions, including cardiovascular disease, diabetes [12], and rheumatoid arthritis [13]. Low levels of physical activity can already be observed in the early stages of the disease [14]. Furthermore, patients with COPD generally walk slower than healthy age-matched controls and are more sedentary [15,16]. The amount and duration of physical activity bouts to perform daily activities decreases with increasing disease severity [17]. Nevertheless, the importance of adequate physical activity levels in patients with COPD cannot be overestimated. A low physical activity level is a strong predictor of poor quality of life and high mortality [14,15]. Consequently, regular physical activity has been shown to reduce the risk of hospital admissions and mortality in patients with COPD [18]. It has also been shown that patients who decreased their activity level had an increased risk of mortality and showed faster disease progression [19]. A recent meta-analysis revealed that any level of physical activity or a reduction of sedentary time is associated with a lower risk of premature mortality in middle-aged and older adults [20].

The characteristic airflow limitation and associated dyspnea of patients with COPD can limit their daily physical activities. This can subsequently lead to physical deconditioning and a further decline in lung function, which can be the start of a deleterious vicious circle of deconditioning [21]. However, the reduced physical activity levels in patients with COPD are not determined by impaired respiratory function alone; other factors such as age, peripheral muscle weakness, hyperinflation, and dyspnea also affect physical activity levels [22]. Alternatively, dog walking and grandparenting have been associated with higher amounts and intensities of physical activity in patients with COPD [23].

All these elements highlight the importance of increasing physical activity levels in patients with COPD. One way of accomplishing this is through comprehensive pulmonary rehabilitation. Rehabilitation belongs to the essential management components in COPD and applied at an early stage of the disease, plays a very important role. A comprehensive rehabilitation program, beyond the physical training components, also includes patient education components on self-management. Patient awareness of current symptom level (either the COPD Assessment Test (CAT) or Modified Medical Research Council (mMRC) scores) and exacerbation frequency assessment have also been found to be very important. Due to the chronic nature of the disease, systematic physical activity, i.e., fitness training on a cycle ergometer or treadmill at a specific intensity, is a key approach to slow down disease progression. Many studies and systematic literature reviews show the beneficial effect of pulmonary rehabilitation in patients with chronic respiratory diseases on exercise capacity [24], lung function [25], respiratory muscle strength [26], and quality of life [27]. The adopted models of pulmonary rehabilitation vary in terms of intensity, duration, and the form of physical activity taken by the patients. Many authors have decided to assess the effect of home rehabilitation, while others have analyzed the impact of early rehabilitation on the hospitalization rate in the next months [28,29,30].

In recent years, increasing attention has been given to evaluating physical activity level as an outcome in patients with COPD [21]. Mantoani et al. carried out a systematic review of 60 intervention studies that evaluated physical activity as an outcome in patients with COPD [31]. The authors concluded that programs combined with coaching interventions and pulmonary rehabilitation programs lasting >12 weeks have the greatest potential to modify physical activity behaviors. Furthermore, it was observed that pulmonary rehabilitation programs do not lead to improved physical activity levels after completion of the program. The majority of patients were unable to maintain an active lifestyle after a rapid increase in exposure to planned supervised physical activity during the rehabilitation program. Thus, it seems that during rehabilitation programs, the focus is mainly on increasing functional exercise capacity and improving symptoms rather than on improving physical activity [6,32].

Besides the increased recognition of the health effects associated with physical (in)activity and the high prevalence of physical inactivity in patients with COPD, the development of technologies and devices that enable objective physical activity assessment in a patient-friendly manner also contributed to the increased interest in physical-activity-related research. Although subjective methods (such as questionnaires) have practical value, wearable accelerometers are likely to provide more accurate information about daily physical activity levels [33]. These devices provide reliable and objective measurements of the duration and intensity of physical activity [34,35]. A combination of subjective and objective methods has also been proposed to obtain a broader assessment of physical activity levels [36]. Wearable accelerometers have, thus, become a standard method of monitoring physical activity in everyday life by measuring acceleration in one, two, or three axes. Triaxial accelerometers have been increasingly used over the years, as they are considered superior to uniaxial accelerometers [37]. Wearable sensors providing user feedback have also been used as a treatment component in numerous physical activity counseling interventions [6]. Additionally, they have been used to assess energy expenditure during walking tests of patients with COPD, where their accuracy of assessment has been positively evaluated [38].

Despite the increased interest in physical-activity-related research, we found a scarcity of literature evaluating physical activity during supervised (weekdays) and non-supervised (weekend) days of a pulmonary rehabilitation program. Therefore, this study used a wearable sensor (SenseWear Armband) to assess physical activity levels during four consecutive days (Friday–Monday) of a 3 week, in-hospital, pulmonary rehabilitation program. We aimed to investigate whether patients have similar physical activity levels during supervised and non-supervised days of a stationary rehabilitation program and whether patients adhere to the rigor of 24 h monitoring. The second objective was to analyze the strengths and weaknesses of such kinds of sensors. We hypothesize that patients present lower physical activity levels during non-supervised days compared to supervised training days.

## 2. Materials and Methods

### 2.1. Participants

The study was conducted among patients who participated in pulmonary rehabilitation at the Specialist Hospital in Glucholazy (Poland). The research enrolled 15 randomly selected patients aged 50–80 years old who met the inclusion criteria. The inclusion criteria were COPD as the main diagnosis and written consent to participate in the study. The exclusion criteria were a main diagnosis other than COPD; pneumonia, tuberculosis, or another respiratory inflammatory disease in all stages and forms; condition after a heart attack; diabetes; state after thoracic and cardiac surgery; heart failure (stage III, IV New York Heart Association (NYHA)); advanced hypertension; diseases and injuries that can impair the function of the musculoskeletal system of transportation; disturbances of consciousness; and psychotic symptoms or other serious psychiatric disorders. The main group characteristics are presented in Table 1. The study adhered to the Declaration of Helsinki [39], and ethical approval was obtained from the Bioethics Committee of the Opole Chamber of Physicians based on Resolution No. 199 of 07 February 2013, and the study was registered in ClinicalTrials.gov (NCT04726384).

### 2.2. Pulmonary Rehabilitation Program

Patients included in the study were qualified for in-patient, 3 week, high-intensity, pulmonary rehabilitation 5 times a week (Monday–Friday, supervised days). During the weekend, patients were encouraged to go for walks and engage in minor physical activity on their own, but during this time they did not take advantage of the organized rehabilitation (non-supervised days). This program has been found to exhibit clinically meaningful improvements in exercise capacity, dyspnea, quality of life, and lung function in patients with COPD [40,41] or lung cancer [42,43]. All procedures were performed under the supervision of a specialist with an M.Phty. degree. The pulmonary rehabilitation program consisted of the following components performed once a day, each for 20–30 min (depending on the task):

- Endurance exercise training on a cycle ergometer to obtain a training heart rate (HR), which was calculated as follows: HR ((max HR − resting HR) × 60%) + resting HR through the use of the results of the 6 min walk test [44], or Borg-rated dyspnea or a fatigue score 4 to 6 (moderate to severe).
- Fitness exercises, coordination, balance exercises, and stretching exercises. Exercises were performed in the following positions: standing; on the knees; and lying on the side, abdomen, and back.
- Specific respiratory exercises: relaxation exercises for breathing muscles, strengthening exercises of the diaphragm with resistance, exercises to increase costal or chest breathing, prolonged exhalation exercise, and chest percussion.
- Inhalation with a 3% NaCl isotonic solution administered with an ultrasonic device.

The rehabilitation program was provided from 8 a.m. to 3 p.m. with a one hour lunch break between 12:30 and 1:30 p.m. During leisure time (after 3 p.m.), patients were encouraged to undertake any physical activity, however, without access to the rehabilitation unit and equipment.

### 2.3. Measurement

The SenseWear armband (Body Media Inc., Pittsburgh, PA, USA) was used to assess physical activity. The device allows for measuring physiological parameters and motion status by using built-in sensors, including the three-axis accelerometer for measuring the number of steps. Using algorithms developed by the producer, the device computes the level of energy expenditure defined in metabolic equivalents of task (METs) and calories during physical activity and rest periods, as well as the total energy expenditure. Additionally, the device counts the total time (min) during lying and during being active (measured when energy expenditure > 3 METs). The device has been considered a reliable source for assessing the physical activity level [45].

**Figure 1:**
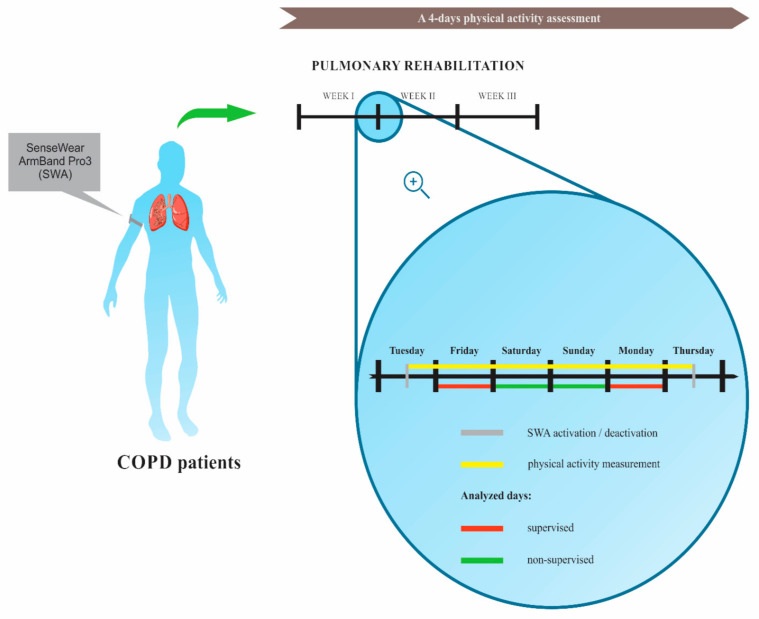
Study flow chart of physical activity assessment.

The group was informed of the purpose of the study and asked to wear the device 24 h a day for the next 4 days (Friday–Monday) excluding bath time, no more than 30 min [46] (Figure 1). Patients received the device on Thursday afternoon and returned it on Tuesday. Patients were also asked to indicate their subjective observations when returning the device at the end of the experiment. For this purpose, we did not use any standardized satisfaction scale; we wanted to explore the strengths and weaknesses of the patients’ feelings.

### 2.4. Statistical Analysis

The sample size was calculated based on the recommendation of the pilot study sample size in the medical field, according to Julious [47] and van Belle [48]: 12 participants were suggested. Considering a 20% drop-out rate, 15 patients were included in the study. Categorical variables were presented as numeric values and percentages, continuous variables as mean ± standard deviation (SD) or median and interquartile range [IQR], where appropriate, according to the Kolmogorov–Smirnov normality test. Differences in the energy expenditure between training days and off days were compared using Mann–Whitney U test or Student’s paired *t*-test. Differences between consecutive days were assessed with Friedman’s ANOVA. All statistical analyses were performed using Statistica 13 software (StatSoft, Cracow, Poland). The statistical significance level was set at α = 0.05.

## 3. Results

The analyzed data were obtained from 13 patients; data from two patients were excluded due to failure to meet recommendations for wearing the armband for 95% of the day (armband off for no more than 30 min a day). We noted that both patients did not meet the requirements to wear the device during non-supervised days. In both cases, the armband was worn around 60% of the time. Results are presented as median [IQR] and mean (±SD).

The analysis of the number of steps per day, the time spent lying as well as undertaking moderate or vigorous physical activity (>3 METs), and the energy expenditure expressed in kcal showed no statistically significant difference between the supervised training days and the non-supervised days off (Table 2).

The mean duration of physical activity > 3 METs was 112 min, which corresponds to the protocol of physical activity during supervised training days. Physical activity on non-supervised days must, therefore, have been generated by physical activities generating an energy expenditure greater than a leisurely walk.

**Figure 2:**
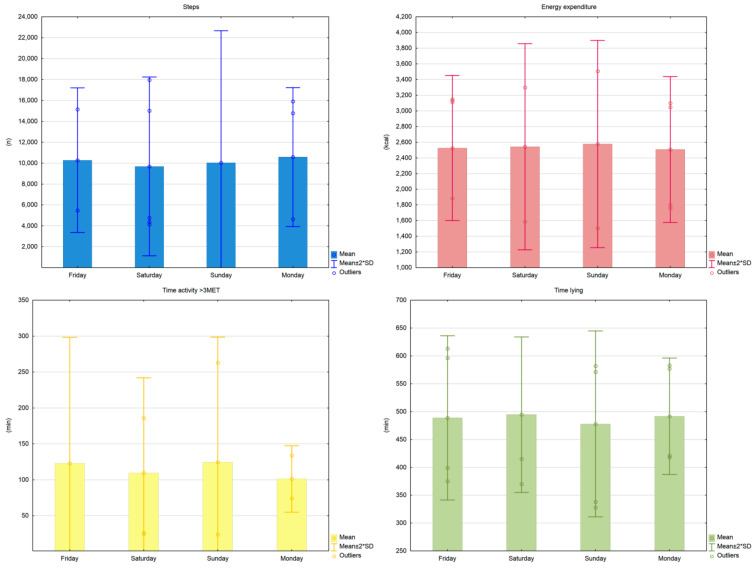
Examined parameters on consecutive days of the study.

Analysis of the results showed no statistically significant differences between the consecutive days of the study for all variables (Figure 2).

## 4. Discussion

This study aimed to investigate the level of physical activity during four days of participation in the pulmonary rehabilitation program of patients with COPD and to compare non-supervised days (weekend) with supervised training days (weekdays). We hypothesized that the patients on non-supervised days will engage in less physical activity. The results showed no significant differences in physical activity levels between supervised and non-supervised days, expressed in energy expenditure (kcal), as well as time spent in moderate physical activity (>3 METs) or spent in a lying position. Thus, the results do not support the hypothesis. Moreover, there were no significant differences in the number of steps between supervised and non-supervised days. These results indicate similar levels of physical activity both on the weekdays and on the weekend. This type of control allows us to assess the involvement of people undergoing rehabilitation at a time when no one supervises them, which in turn is important in the context of the effectiveness of the entire treatment process. Thus, our results are of great clinical importance, it has been shown that modifications to patient behavior that enhance adherence to health-enhancing patient behavior and increase activity levels in everyday life [49] are key factors to maintaining the improved physical capacity achieved through participation in pulmonary rehabilitation. To our best knowledge, to date, this is the first study evaluating energy expenditure during two distinct activities: supervised activity during the pulmonary program and non-supervised days in patients with COPD during a 3 week, in-hospital, rehabilitation program.

Lahham et al. compared levels of physical activity during center- and home-based pulmonary rehabilitation in people with COPD using the SenseWear Armband device [50]. Differences in time spent in total physical activity (≥1.5 METs), time spent in moderate to vigorous–intensity physical activity (≥3 METs), and steps were compared. Home rehabilitation participants engaged in a mean of 310 (199–328) min per day of physical activity (29% moderate- to high-intensity physical activity) when compared to center-based rehabilitation participants who spent a mean of 300 (204–370) min per day (28% moderate- to high-intensity physical activity, *p* = 0.98). The daily number of steps did not differ between groups; home rehabilitation: 5232 [2067–7718], while for in-center rehabilitation, it was 4049 [1983–6040], *p* = 0.66). In our study, we noted a higher number of steps taken by patients. However, it is difficult to compare the time spent on physical activity because we assumed different levels of minimum energy expenditure, in our study ≥3 METs, while Lahham et al. [50] used ≥1.5 METs.

Ward et al. utilized a different type of activity monitor in their study, i.e., the Fitbit Zip. It was used in the study to measure the number of steps during a 6 week pulmonary rehabilitation intervention. The number of total steps taken per day between week 1 and week 6 of the intervention increased by 20% (week 1: 3565 [95% confidence interval (CI) 2779–4351] vs. week 6: 4447 [95% CI 3333–5561] steps/day, *p* = 0.036), whereas the number of steps taken during the recommended pulmonary rehabilitation exercise increased by 56% (week 1: 595 [95% CI 397–793] week 6: 927 [95% CI 599–1256] steps per day, *p* = 0.009) [51]. Geidl et al. analyzed a sample of 326 patients with COPD and their level of physical activity and time spent sitting during the 8 days before the pulmonary rehabilitation program using the ActiGraph wGT3X device [52]. The study group was divided into four subgroups based on time spent sitting and physical activity intensity. The daily step counts in that study ranged from 2749 (sedentary non-movers) to 5649 (sedentary occasional movers), to 7866 (sedentary movers), to 11,045 (sedentary exercisers). All four subgroups had a long sedentary daily routine (7.5–10.75 h). The mean age of the study group was 58 years, and most of the subjects were professionally active, most probably because of this, the daily step count results met the recommendations for patients with COPD who need to achieve >4580 steps per day [53] to avoid severe physical inactivity. The results show that patients with COPD have different levels of physical activity in free-living conditions. However, most patients with COPD spend a significant and unhealthy portion of their daily lives engaging in sedentary behavior.

A non-supervised method for stimulating patients with COPD to increase their physical activity levels in free-living conditions was presented by the Urban Training™ Study Group [23,54,55]. First, urban walking trails of different intensities and in different types of public spaces (e.g., beach or park) were designed and validated [54]. Afterward, a randomized controlled trial of 407 patients with COPD was performed, in which the intervention group was advised to walk on the developed urban trails but without any supervision. These patients furthermore received a pedometer and personalized calendar to monitor their physical activity, in combination with other behavioral strategies for increasing their physical activity levels (i.e., physical activity brochure, website, phone text messages, walking groups, and a phone number). This intervention was implemented for 12 months and proved to be efficacious in increasing physical activity levels, quantified by the amount of steps per day over the course of a week, in patients with COPD [55].

Based on the above-mentioned studies, the first attempts have already been made to assess the accurate estimation of physical activity levels of patients with COPD in either a supervised or non-supervised setting. However, another aspect seems to be the development of technology for this kind of study. Regarding the second objective of the study, i.e., the strengths and weaknesses of such kinds of sensors, the subjective acceptance by patients of such a monitoring system was noted. Patients indicated in their final reports that they were unaware of wearing the sensor, except when they over-tightened the device on the attachment strap after bathing. However, the authors noted a high frequency of returning dirty devices. In our opinion, this indicates that patients did not wash the devices, although at the beginning of the study participants were informed about the possibility of washing with warm water the part of the sensors that are directly attached to the skin.

McNamara et al. evaluated the comfort of the SenseWear armband on a group of patients with COPD [35]. Results indicate that adverse effects may occur during the use of the device, most commonly in the form of skin itching, redness, and bruising. Moreover, 17% reported that the device was uncomfortable to wear at night, and 11% reported that it was uncomfortable to wear during the day. Despite this, compliance in wearing the SenseWear armband over 7 days was very high in this study (92%). Similarly, a one week observational study of patients with COPD reported no issues with using the SenseWear armband to provide contextual information about physical activity and sleep over the course of 7 days [56]. In a prospective study at three Northern European sites, the SenseWear armband was used to assess physical activity levels over 6 consecutive days in 134 patients with COPD and 46 controls. The authors defined a valid measurement period as a wearing time higher than 22 h per day, on at least 5 days. Excellent compliance with wearing the SenseWear armband was reported, with at least 94% of the patients in the three different sites having a valid measurement period [57].

An international team of investigators sought to validate six physical activity monitors in patients with COPD against a gold standard of indirect calorimetry in the form of oxygen uptake data from a portable metabolic system. The study used single-axis accelerometers: Kenz Lifecorder Plus and Actiwatch, and triaxial accelerometers: RT3, ActiGraph GT3X, DynaPort^®^ MiniMod, and SenseWear Armband. The study concluded that triaxial activity monitors were the best monitors to assess intensity physical activity for patients with COPD [58]. Patel et al. suggest that the SenseWear Pro armband may be a useful tool for assessing physical activity levels during therapeutic interventions [38]. Cavalheri et al. found it useful for assessing total energy expenditure during activities of daily living in patients with COPD [59]. Our observations support this conclusion. We noted 87% adherence to the study, where it was possible to obtain more than 95% of patient monitoring on 4 consecutive days. The individuals who were lost returned the device within the designated timeframe, but the device wear rate was below the accepted threshold. Visual assessment of the charts of these individuals indicated that the device was usually left in place for several hours, usually the evening hours (5–10 pm). In the authors’ speculations, it seems possible that these actions were intentional, as there were pieces of information to hospital staff that patients attended “informal” evening meetings. As an alternative to the SenseWear armband, the Polar A300™ can be worn as a wrist device similar to a watch. Boeselt et al. compared the two devices in regards to the number of steps, burned calories, daily activity time, and metabolic equivalents in patients with COPD over 3 days of daily life [60]. Data analysis over 3 days showed that 90% of the steps (95% CI over/under the means between Polar A300™ and SWA −4223–1887), 100% of the calories (95% CI −2798–1887), 90% of the daily activity data (95% CI −12.32–4065), and 95% of the MET (95% CI −3.11–2.75) were within the limits of agreement. The A300™ device is no worse at assessing physical activity time, step count, and calorie consumption in patients with COPD compared to SenseWear Armband.

Technological advances have, furthermore, allowed the combination of measurements of physical activity with other (physiological) measurements, such as heart rate. Joosen et al. implemented such a mobile health system, consisting of a smartphone and heart rate monitor, in a care home setting for 10 weeks [61]. Triaxial accelerometry data from the smartphone were converted into interpretable activity (e.g., steps per hour, time walking, walking distance) and stride (e.g., stride duration, stride speed, stride displacement) features, while heart rate measurements were converted into interpretable heart rate features (e.g., median heart rate, minimal heart rate, time constant of heart rate increase). Participants received weekly feedback about their activity and heart rate features. The implementation of this mobile health system was associated with increased physical activity levels during the first 5 weeks of the study, after which physical activity levels starting declining again. In addition, the calculated features were converted into a fitness score, which could predict the outcome of more labor-intensive exercise tests.

More recently, the combination of physical activity and heart rate measurements has been used to address the current COVID-19 pandemic. Quer et al. were able to discriminate between COVID-19 symptomatic positive and negative cases (area under the curve of 0.80) by combining self-reported symptoms with measurements of physical activity, sleep and heart rate [62]. Natarajan et al. obtained an area under the curve of 0.77 for the prediction of illness on a specific day, based on measurements with a Fitbit for that day and the preceding 4 days [63]. Mishra et al. observed that 26 out of 32 individuals who were infected with COVID-19 had alterations in their daily steps, time asleep, or heart rate [64]. These studies show that measurements with wearable sensors could be used for the early detection of COVID-19.

To the best of our knowledge, our study is the first to explore the weekday-to-weekend physical activity level among patients with COPD during in-hospital pulmonary rehabilitation. Although this study provides encouraging results, we recognize that some limitations should be considered. Firstly, the research included a small study group. Secondly, the number of observation days could be extended. Investigating only one weekend may introduce bias in the results since physical activity may have been influenced by, for example, good weather conditions. Finally, energy expenditure was assessed using a commercial activity monitor and stimulated estimation of energy expenditure using machine learning on multimodal data. To accurately measure the energy expenditure, there are methods such as doubly labeled water and direct and indirect calorimetry, but their cost and practical limitations make them suitable only for stationary research and professional sports.

## 5. Conclusions

Interest in objective measures of physical activity in patients with COPD due to the close relationship between physical activity levels and exercise tolerance, disease symptoms, disability incidence, and mortality continues to rise. Therefore, it seems beneficial to use available physical activity monitors in patients with COPD, as measurable parameters provide feedback that may increase the patient’s motivation to be active to achieve health benefits. Portable, lightweight, skin sensors mounted on the arm or wrist appear to provide adequate comfort and meaningful measurements to monitor and modify patient behavior to enhance adherence to health-enhancing patient behavior and increase activity level in everyday life.

## Figures and Tables

**Table 1:** Group characteristic.

Variables	Mean (SD)
Age (years), mean (SD)	63.8 (9.1)
Female, n (%)	7 (54%)
Height (cm), mean (SD)	168 (9.3)
Weight (kg), mean (SD)	79.7 (15.6)
BMI (kg/m^2^), mean (SD)	28.1 (4.3)
Smokers, n (%)	2 (15%)
FEV1 (%), means (SD)	78.2 (14.9)

FEV1: forced expiratory volume for 1 second, SD: standard deviation, BMI: body mass index.

**Table 2:** Results of the study.

Variable		Training Days (n = 26)	Off Days (n = 26)	*p*
Steps (n)	Median [IQR]	9153 [7744–12,524]	8421 [5668–12,552]	0.57 *
	Mean (SD)	10,428 (3323)	9859 (5287)	
Active time (min)	Median [IQR]	110 [76–128]	119 [65–140]	0.75 *
	Mean (SD)	112 (64)	117 (76)	
Time lying (min)	Median [IQR]	492 [444–526]	480 [449–547]	0.77 **
	Mean (SD)	490 (63)	486 (76)	
Energy expenditure (kcal)	Median [IQR]	2616 [2089–2899]	2491 [2011–2812]	0.58 **
	Mean (SD)	2517 (455)	2560 (646)	

* According to Wilcoxon test, ** according to t-Student test. IQR: interquartile range.

## Data Availability

The data presented in this study are available on request from the corresponding author.
